# The Production of Standardized Samples with Known Concentrations for Severe Acute Respiratory Syndrome Coronavirus 2 RT-qPCR Testing Validation for Developing Countries in the Period of the Pandemic Era

**DOI:** 10.1155/2021/5516344

**Published:** 2021-08-03

**Authors:** Hoang Quoc Cuong, Nguyen Duc Hai, Hoang Thuy Linh, Nguyen Trung Hieu, Nguyen Hoang Anh, Tran Ton, Tran Cat Dong, Vu Thanh Thao, Do Thi Hong Tuoi, Nguyen Duc Tuan, Huynh Thi Kim Loan, Nguyen Thanh Long, Cao Minh Thang, Nguyen Thi Thanh Thao, Phan Trong Lan

**Affiliations:** ^1^Microbiology and Immunology Department, Planning Division, Medical Testing and Calibration Centers, Medical Analysis Department, Pasteur Institute in Ho Chi Minh City, Vietnam; ^2^Faculty of Pharmacy, University of Medicine and Pharmacy at Ho Chi Minh City, Vietnam

## Abstract

**Background:**

Severe acute respiratory syndrome coronavirus 2 (*SARS-CoV-2*) has caused a pandemic of pneumonia spreading around the world, leading to serious threats to public health and attracting enormous attention. There is an urgent need for sensitive diagnostic testing implementation to control and manage *SARS-CoV-2* in public health laboratories. The quantitative reverse transcription PCR (RT-qPCR) assay is the gold standard method, but the sensitivity and specificity of SARS-CoV-2 testing are dependent on a number of factors.

**Methods:**

We synthesized RNA based on the genes published to estimate the concentration of inactivated virus samples in a biosafety level 3 laboratory. The limit of detection (LOD), linearity, accuracy, and precision were evaluated according to the bioanalytical method validation guidelines.

**Results:**

We found that the LOD reached around 3 copies/reaction. Furthermore, intra-assay precision, accuracy, and linearity met the accepted criterion with an RSD for copies of less than 25%, and linear regression met the accepted *R*^2^ of 0.98.

**Conclusions:**

We suggest that synthesized RNA based on the database of the NCBI gene bank for estimating the concentration of inactivated virus samples provides a potential opportunity for reliable testing to diagnose coronavirus disease 2019 (COVID-19) as well as limit the spread of the disease. This method may be relatively quick and inexpensive, and it may be useful for developing countries during the pandemic era. In the long term, it is also applicable for evaluation, verification, validation, and external quality assessment.

## 1. Introduction

A novel coronavirus that belongs to the Coronaviridae family has caused an outbreak of pneumonia spreading around the world, leading to serious threats to public health and attracting enormous attention [1, 2]. While waiting for the distribution of a vaccine across countries, in particular developing countries, or for the approval of new medicines, quantitative reverse transcription (RT-qPCR) is a key solution to combating this pandemic. Early detection of severe acute respiratory syndrome coronavirus 2 (*SARS-CoV-2*) may be of importance for halting the spread of this disease in the community. Most diagnostic assays being applied for the diagnosis of coronavirus disease 2019 (COVID-19) infections involve the RT-qPCR assay [3, 4], which is obligatory, especially in the treatment and isolation of early-infected patients [5]. Furthermore, there is an urgent need for sensitive diagnostic testing implementation to control and manage *SARS-CoV-2* in public health laboratories [6]. Up to now, the World Health Organization (WHO) has listed only 17 countries that have developed laboratories and protocols for the detection of *SARS-CoV-2* [7]. Besides, WHO endorsed nations who have no testing capacity, and national COVID-19 laboratories with inadequate experience of SARS-CoV-2 testing are stimulated to send specimens to the WHO reference laboratory for confirmation [7].

Although a number of approaches are available to detect the SARS-CoV-2 viral genome, the sensitivity and specificity of *SARS-CoV-2* testing depend on a variety of variables, such as the position of clinical specimens, the low viral load of the patient, the intermittent shedding, and the difference between multiple detection kits by producers [8, 9]. The majority of COVID-19 symptoms resemble those of the common flu or the cold. It is therefore critical that infected individuals be diagnosed early and accurately if this fatal disease is to be prevented from spreading widely. The identification in the early stages of the COVID-19 patients allows doctors to assist before serious complications have developed [10]. Furthermore, with a scarcity of chemical reagents for SARS-CoV-2 diagnosis and an increase in SARS-CoV-2 cases, the development of standardized specimens with known concentrations for validation is critical [11, 12]. Here, we synthesized RNA based on the genes published to estimate the concentration of inactivated virus samples for screening programs for COVID-19.

## 2. Materials and Methods

### 2.1. Sequence of the SARS-CoV-2 Virus and Synthesis of Artificial DNA

In this study, the sequence of the *SARS-CoV-2* virus was used based on the database of the NCBI gene bank (GenBank NC_045512.2). We used the primer and probe sequences that were published in the previous study [13]. SARS-CoV-2's E gene from nucleotide no. 26132 to 26529 was as follows: ACACAATCGACGGTTCATCCGGAGTTGTTAATCCAGTAATGGAACCAATTTATGATGAACCGACGACGACTACTAGCGTGCCTTTGTAAGCACAAGCTGATGAGTACGAACTTATGTACTCATTCGTTTCGGAAGAGACAGGTACGTTAATAGTTAATAGCGTACTTCTTTTTCTTGCTTTCGTGGTATTCTTGCTAGTTACACTAGCCATCCTTACTGCGCTTCGATTGTGTGCGTACTGCTGCAATATTGTTAACGTGAGTCTTGTAAAACCTTCTTTTTACGTTTACTCTCGTGTTAAAAATCTGAATTCTTCTAGAGTTCCTGATCTTCTGGTCTAAACGAACTAAATATTATATTAGTTTTTCTGTTTGGAACTTTAATTTTAGCCATGGCAG.

**Figure 1:**
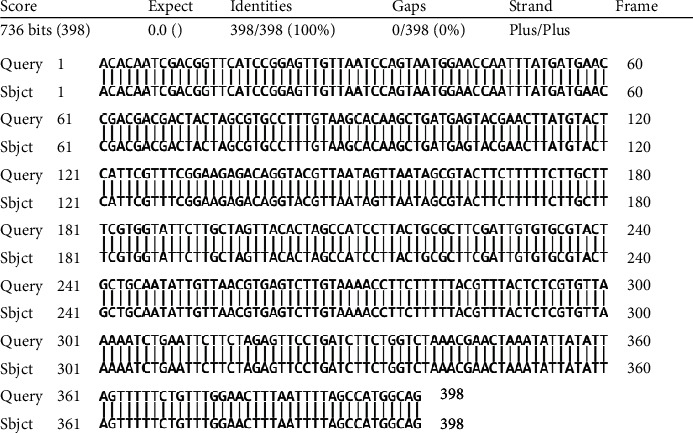
Alignment results of the synthetic E gene sequence compared with the E gene sequence on GenBank.

**Figure 2:**
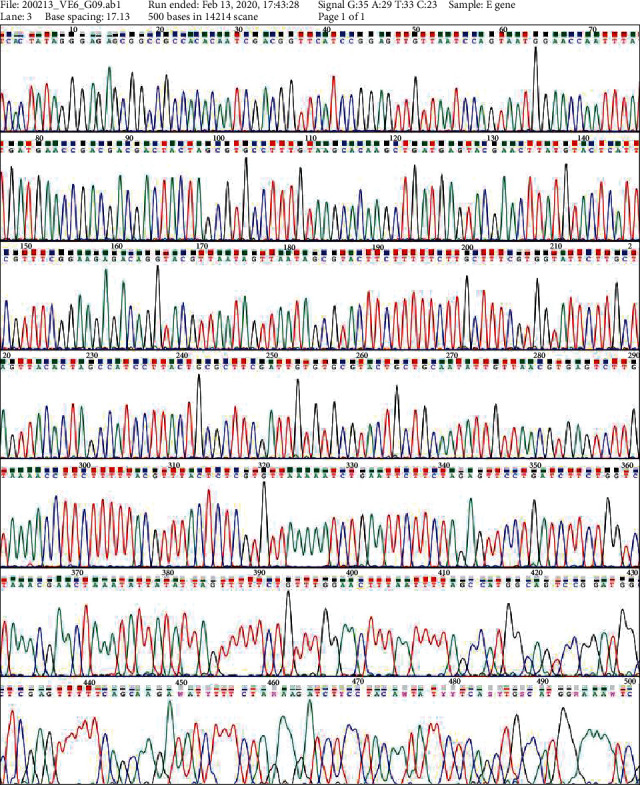
E gene sequencing data.

DNA is synthesized in vitro by assembling single-stranded oligonucleotide fragments of 60-70 nucleotide length by the catalysis of Phusion™ High-Fidelity DNA Polymerase (2 U/*μ*L) (Thermo Fisher Scientific) using the PCR technique. After assembly, product inspection was performed by 2% gel agarose electrophoresis. After assembly, the size of the DNA was determined by electrophoresis on 2% agarose gel and then transformed into the pJET1.2 plasmid (Phu Sa Corp), and the transformation was screened. The transformed plasmid was extracted and sequenced by using the E gene synthesized in vitro. The sequencing results showed that the E gene was synthesized to have the same sequence as the originally designed E gene. We used the MAFFT online server with the default parameters to align the SARS-CoV-2 genome sequences [14], and the complete genome sequence from the NCBI gene bank (GenBank NC_045512.2) was used as a reference genome. The result of aligning the E gene sequence with the E gene sequence on GenBank showed that the two sequences are similar to each other, in which query covers 100% and has an E value of 0.0 (Figure 1). E gene sequencing data is illustrated in Figure 2.

### 2.2. Preparation of DNA-Carrying Plasmids (Recombinant DNA)

The synthesized length of the gene fragments was connected to the pJET 1.2 plasmids using the cloning system (Phu Sa Corp). The recombinant plasmid was inserted into *Escherichia coli* (MAX Efficiency DH5*α* (Life Technologies, Carlsbad, CA; chemically competent, ~10^9^ colony-forming unit or CFU/*μ*g pUC19)) on Luria-Bertani agar plates (Thermo Fisher Scientific, consisting of 10 g L^−1^ tryptone (peptone from casein), 5 g L^−1^ yeast extract, and 5 g L^−1^ sodium chloride) containing 100 *μ*g/mL ampicillin. Five colonies on a pJET 1.2 plasmid were chosen for checking the transformation process by PCR with specific primers (pJET1.2, Phu Sa Corp; pJET1.2.Fw (5′-d(CGACTCACTATAGGGAGAGCGGC)-3′) and pJET1.2.Rv primers (5′-d(AAGAACATCGATTTTCCATGGCAG)-3′). The modified vector-carrying strains were grown on Luria-Bertani broth overnight and recovered with the FavorPrep Plasmid Extraction Mini Kit (Favorgen, Biotech Corp) according to the manufacturer's recommended procedures. The transformation results were confirmed by genetic sequencing with specific primers and plasmid pJET1.2 primers, using the Sanger sequencing technique on the Applied Biosystems 3130 device.

### 2.3. RNA Synthesis In Vitro

The recombinant plasmid was extracted and straight-lined with 2 restriction enzymes NotI (NotI (10 U/*μ*L), Thermo Fisher Scientific) and Kpn2I (BspEI (10 U/*μ*L), Thermo Fisher Scientific) following the manufacturer's instructions.

Straight-line plasmid fragments after treatment with a restriction enzyme were transcribed into RNA by the catalysis of the enzyme T7 RNA polymerase (Ambion™ T7 RNA Polymerase, cloned, 200 U/*μ*L, Thermo Fisher Scientific) following the instructions of the manufacturers. The RNA was purified and preserved in diethyl-pyrocarbonate water. The RNA was then tested on a 1.2% agarose gel electrophoresis system using a 0.5X TBE buffer.

The quantity of pure RNA product (ng/*μ*L) was determined in triplicate on a NanoDrop by measuring absorbance at 260 nm with a spectrophotometer (NanoDrop™ 2000/2000c Spectrophotometer, Thermo Fisher Scientific). Based on the size and nature of the target RNA structure, the yield for each control was calculated using the Avogadro conversion factor (6, 022 × 10^23^): number of copies = (weight (ng) × 6, 022 × 10^23^)/(length × 330 × 10^9^).

### 2.4. Culture

The *SARS-CoV-2* virus was isolated from throat swab samples of patients with COVID-19 infection [15]. In brief, these specimens were placed in a viral transport medium and refrozen; then, the virus was subsequently grown in Vero E6 cells (ATCC#C1008) in the Pasteur Institute in Ho Chi Minh City, Vietnam. Vero E6 cells were cultured in Dulbecco's minimal essential medium (DMEM, D1145, Sigma-Aldrich, US) supplemented with 10% heat-inactivated fetal bovine serum (FBS, Gibco, Thermo Fisher, US) and antibiotics [16]. On the isolation day, 50 *μ*L serum-free DMEM was added into columns 2-12 of a 96-well culture plate. 100 *μ*L clinical specimens were pipetted into column 1 and then serially diluted 2-fold across the plate. After trypsinization (Gibco Trypsin-EDTA, Thermo Fisher, US), Vero E6 cells were suspended in DMEM supplemented with 10% FBS, 200 IU/mL penicillin-streptomycin (Sigma-Aldrich, US), and 5 *μ*g/mL amphotericin B (Sigma-Aldrich, US) with 2.5 × 10^5^ cells/mL. 100 *μ*L of cell suspension was directly added to the clinical swab dilutions and gently mixed by pipetting. The inoculated cultures were grown in a humidified atmosphere of 5% CO_2_ at 37°C. The cytopathic effects (CPE) were observed in cells after incubation for three days. Standardized plaque assays were applied to *SARS-CoV-2* according to the *SARS-CoV* and *MERS-CoV* protocols [17, 18].

The SARS-CoV-2 strain, SARS-CoV-2/human/VNM/nCoV-19-02S/2020, was used in this study, and the genome sequence of the strain was deposited in GenBank (MT192773.1). In brief, the genome sequence length was 29,890 bp with no gaps and a high coverage of 1,897x. This strain was of the type betacoronavirus B and has 99.98 percent nucleotide-level similarity isolated in Wuhan and >90.56 percent pangolin-isolated *SARS-CoV* similarity. Four mutations have been identified as nonsynonymous mutations, such as G8388A (asparagine serine), A8987 T (phenylalanine isoleucine), and A8987 T (phenylalanine isoleucine) [9, 19].

### 2.5. RT-qPCR for *SARS-CoV-2*

RNA synthesis was placed into a collection tube with 150 *μ*L of virus preservation solution. In brief, 40 *μ*L of cell lysate was transferred into a collection tube, followed by a vortex for 10 seconds. After standing at room temperature for 10 minutes, the collection tube was centrifuged at 1000 rpm/min for 5 minutes. The suspension was used for a quantitative reverse transcription PCR (RT-qPCR) assay of *2019-nCoV* RNA. The E target gene was amplified and tested during the RT-qPCR assay with the forward primer 5′-ACAGGTACGTTAA TAGTTAATA GCGT-3′ and reverse primer 5′-ATATTGCAGCAGTACGCACACA-3′ and the probe 5-′FAM ACACT AGCCATCCTTACTGCGCTTCGBBQ-3′. All oligonucleotides were synthesized and provided by TIB-Molbiol (Berlin, Germany).

**Figure 3:**
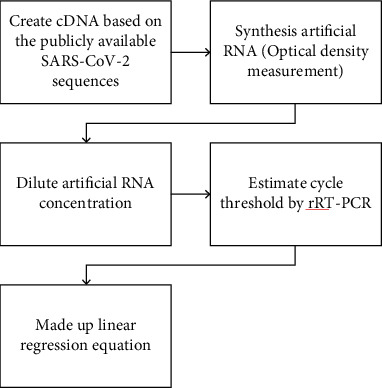
The development of a linear regression equation from artificial RNA.

**Figure 4:**
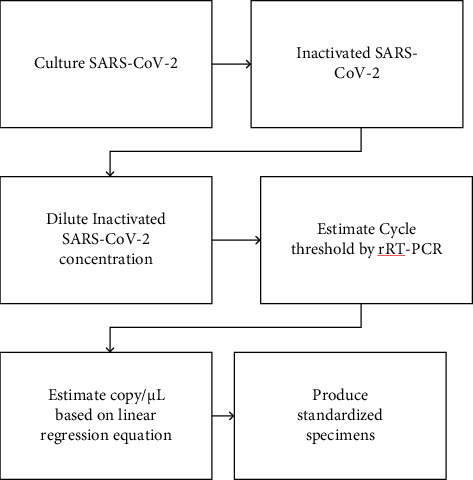
The process of producing standardized specimens of known concentration based on the linear regression equation.

RT-qPCR was performed in triplicate on the LightCycler*®* 480 System using a 2019-nCoV nucleic acid detection kit according to the manufacturer's protocol (TIB-Molbiol reagents). The reaction mixture contained 5 *μ*L of RNA, 12.5 *μ*L of 2× reaction buffer provided with the Superscript III one-step RT-qPCR system with Platinum Taq Polymerase (Invitrogen, Darmstadt, Germany; 0.4 mM of each deoxynucleotide triphosphate (dNTP) and 3.2 mM magnesium sulfate), 1 *μ*L of reverse transcriptase/Taq mixture from the kit, and 0.4 *μ*L of a 50 mM magnesium sulfate solution (Invitrogen) [13]. RT-qPCR was performed under the following conditions: incubation at 55°C for 03 minutes and 95°C for 30 seconds, 45 cycles of denaturation at 95°C for 03 seconds, and extending and collecting the fluorescence signal at 60°C for 12 seconds. A cycle threshold value (Ct value) of 40 or more was defined as a negative test [9, 20]. The development of a linear regression equation from artificial RNA and the process of producing standardized specimens of known concentration based on a linear regression equation are shown in Figures 3 and 4.

### 2.6. Analysis

Data were entered using EpiData version 3.1 (EpiData Association, Odense, Denmark, 2005), and all statistical analyses were carried out using Stata version 13.0 (StataCorp, TX, 2013). The obtained sequences (E gene) were manually edited with Chromas Lite 2.1.1 (Technelysium Pty Ltd., South Brisbane, AU). The results were summarized using mean and standardized deviation (SD) and the relative standard deviation (RSD) for continuous variables. Linear regression analysis was performed to estimate the linear regression equation. In this study, the percentage of RSD for accuracy, intermediate precision, and repeatability was less than 25% [21–23].

### 2.7. Ethics Approval

The study protocol has been reviewed and ratified by the Pasteur Institute in Ho Chi Minh City Institutional Review Board (reference number: 433/XN-PAS).

## 3. Results

### 3.1. Estimated Concentration of RNA

The RNA after in vitro synthesis was purified, and optical density was measured on the NanoDrop device to calculate the RNA concentration. Photometric results showed that the ratio of A260/A280 was 2.1; thus, the RNA was purified. The mean RNA concentration of the three replicates was 109.43 ± 0.74 ng/*μ*L. We calculated the number of copies/*μ*L at 4.83 × 10^11^ (Table 1). The artificial RNA, after determining the number of copies, is diluted to the appropriate concentration to investigate the detection limit, accuracy, and precision.

### 3.2. Precision and Accuracy of Ct Values and Copy Concentration of Synthesized RNA

**Figure 5:**
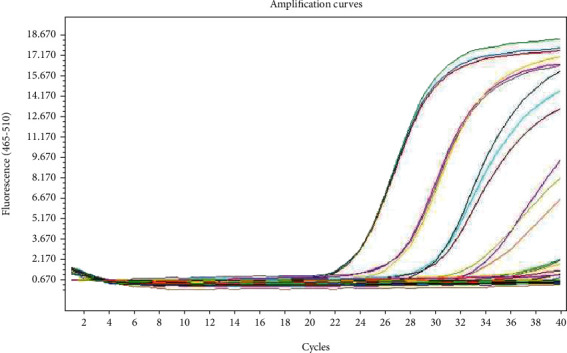
Ct values of synthesized RNA.

In the present study, we tested a dilution series of nine replicates for synthesized RNA per concentration. The Ct value ranged from 22.4 to 23.69, and the number of copies was 48,300 copies/*μ*L at the level of 10^4^ dilutions. When dilution tends to be small, the Ct values are more likely to increase. There was quite consistent fluorescence at 22, 24, and 28 cycles per concentration (Figure 5).

### 3.3. The Precision of the Ct Values in Each Dilution Concentration of Synthesized RNA

#### 3.3.1. Repeatability

Intra-assay precision was determined from assay results in each concentration run. The RSD for log copies of each concentration was 0.77-8.33%, which reached the accepted criterion of RSD < 25% (Table 2) [23, 24].

#### 3.3.2. Intermediate Precision

Similarly, intra-assay precision was performed from assay results on four different runs and on three different days. The RSD for log copies of each concentration was 2.80-13.30%, which meets the accepted criterion of an RSD of less than 25% (Table 3) [23, 24].

### 3.4. Accuracy of Ct Value in Each Dilution Concentration of Synthesized RNA

Because the concentration of synthesized RNA was 48,300 copies/*μ*L, we diluted it to 80% and 120% of the original concentration. RT-qPCR was then repeated seven times on different days. The percentage of RSD for log copies was 1.01-2.70% with an RSD of <25% [23]. The results also exhibited that the synthesized RNA specimens had a good extraction efficiency (Tables 4 and 5).

**Figure 6:**
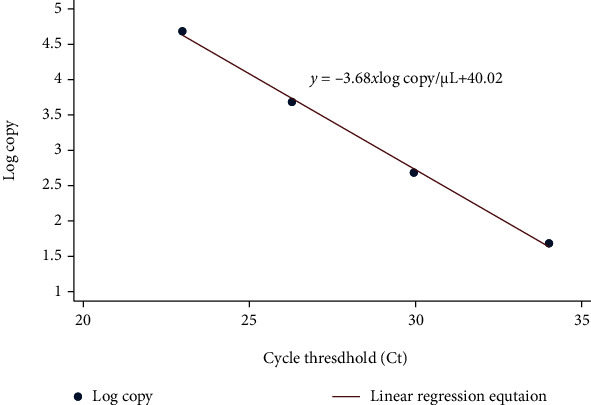
The linear regression based on the Ct value and log copy.

In this study, the linearity was created based on the Ct value versus log copy. The linear regression was obtained from five independent assays performed on different days. We estimated the linear regression equation with *Y* = −3.68*x*log copy/*μ*L + 40.02. The *R*^2^ of the standard linear equation reached 0.998, which meets the accepted criterion of *R*^2^ > 0.98 (Figure 6) [23, 24].

### 3.5. Estimated Concentration of RNA Extracted from the SARS-CoV-2 Virus

RT-qPCR was used in a dilution series of ten replicates run on three different days to estimate the Ct values based on the standard curves above to estimate the concentration of RNA extracted from the SARS-CoV-2 virus isolated from Vero E6 cells. The percentage of RSD for log copies was 0.54-14.61% with an RSD for log copies of <25% (Table 6) [23, 24].

### 3.6. Limit of Detection (LOD)

By establishing a standard curve, linearity was also confirmed and the LOD was calculated (~3 copies/reaction). In this study, intra-assay precision, accuracy, and linearity met the acceptance criteria with an RSD for log copies of less than 25% (Table 7) [23, 24].

## 4. Discussion

The current study reported the process of developing standardized specimens of known concentration, which were RNA extracted from the *SARS-CoV-2* virus isolated, for evaluation, verification, validation, and external quality assessment. Standardized specimens are produced according to the international conference on harmonization guidelines for bioanalytical method validation [21–23, 25]. After creating the standard curve, RNA extracted from the inactivated virus was processed in a RT-qPCR system to estimate the standardized specimens at a known concentration. We found that the LOD reached 3 copies/reaction, and intra-assay precision, accuracy, and linearity met the accepted criterion with RSD for log copies of less than 25% [23, 24]. Besides, linear regression meets the accepted *R*^2^ of 0.98 [21–24]. It could help to assess the reliability of SARS-CoV-2 testing in order to improve the testing capacity of COVID-19 screening strategies [9]. This method may be relatively quick and inexpensive, making it useful for developing countries during the pandemic.

The *SARS-CoV-2* (E, N, and RdRP gene detection) test is a high-throughput RT-qPCR technique for the qualitative identification of *SARS-CoV-2* nucleic acid in nasopharyngeal, nasal, and oropharyngeal swab samples from subjects suspected of COVID-19 [10]. In the current tests, the primer and probe sets are designed to detect three regions of the *SARS-CoV-2* single-stranded RNA genome [10]. More specifically, the RdRP (RNA-dependent RNA polymerase) and N genes (a nucleocapsid protein) are *SARS-CoV-2* specific, whereas the E gene (an envelope protein) is Sarbecovirus specific [10, 26]. Chu et al. suggested the ORF1b gene for confirming the results and the N gene for screening [27], while Corman et al. recommended confirming the test results with the RdRp gene assay and using the E gene assay as the first-line screening tool [13]. Although our study only developed standardized specimens of known concentration based on the E gene, developing quick and accurate COVID-19 screening methods will also assist in identifying negative people and avoiding unnecessary COVID-19 quarantines, which have had a severe influence on social life and resulted in a significant economic crisis [10].

To detect the SARS-CoV-2 viral genome, a variety of approaches are available, including PCR-based SARS-CoV-2 detection (reverse transcription-quantitative PCR, reverse transcription digital PCR, and current isothermal amplification methods) and nonconventional methods (genome sequencing, clustered regularly interspaced short palindromic repeat-based COVID-19 detection, and nanoparticles). Of note, the sensitivity of the droplet digital PCR was found to be equal to or greater than that of the RT-qPCR [28, 29]. These approaches, however, are not equivalent to RT-qPCR tests in terms of cost, sensitivity, or specificity [30]. Until now, the gold standard technique is the molecular diagnosis of SARS-CoV-2 using the RT-qPCR assay [13, 31]. However, RT-qPCR assays depend on the similarity of SARS-CoV-2 to SARS-CoV, collecting time and location of the specimens [32]. Consequently, false-positive RT-qPCR results have been reported in the SARS-CoV-2 diagnosis output in recovery patients and asymptomatic infected patients [33]. RT-qPCR assays necessitate the use of relatively expensive instruments as well as highly trained personnel. Thus, these provisions limit diagnostic capacity expansion in several countries [30]. Taken together, the validation of rapid diagnostic tests for COVID-19 should be a priority for diagnosis and follow-up of patients both in the hospital and in the community, allowing us to detect cases early and isolate patients and close contacts rapidly. Access to reliable rapid diagnostic tests could improve the pressure on laboratories and enlarge the testing capacity to meet the most urgent medical and public health needs [13].

The *SARS-CoV-2* pandemic posed a serious threat to human health. However, shortages of chemical reagents and healthcare workers are restraining testing capacity from the growing demand for COVID-19 diagnostics around the world, especially in resource-limited settings [9, 13]. However, strategies from WHO emphasized the vital role of early testing of suspected cases to halt virus spread and emphasized that the need for reliable assays to detect and laboratory confirm cases early [34] is therefore essential to evaluate the reliability of the RT-qPCR testing. While waiting for a vaccine to be distributed to every country, especially developing countries, or medicine to be approved, RT-qPCR is an essential solution in the fight against this pandemic. Standardized specimens of known concentration will contribute to improving the testing capacity and support screening, earlier diagnosis of infection, and isolation. In the long-term, standardized specimens of known concentrations are capable of evaluation, verification, validation, and external quality assessment.

In the current study, we developed standardized specimens and followed the guidelines on bioanalytical method validation [21–23, 25]. Our study has several limitations. First, we only created standardized specimens of known concentration using the E gene, and our findings were unable to achieve a broad dynamic range [27]. Second, we only identify the purity of RNA based on UV absorbance, which is not strong enough for the quantification of the results, because results may be influenced by the impurity of the samples. However, RNA is pure (the ratio A260/A280 was 2.1).

## 5. Conclusions

In summary, we synthesized RNA from the database of the NCBI gene bank to estimate the concentration of inactivated virus samples and provided a potential opportunity for reliable testing to diagnose COVID-19. From there, standardized specimens of known concentrations contribute to improving the testing capacity and supporting early diagnosis of infection as well as limiting the spread of the disease. This method may be relatively quick and inexpensive, and it may be useful for developing countries during the pandemic era. In the long term, standardized specimens of known concentrations are applicable for evaluation, verification, validation, and external quality assessment.

## Figures and Tables

**Table 1:** Characteristics of synthesized RNA.

Gene	Length	A260/280	A260/230	Concentration (copy/*μ*L)	Numbers (copy/*μ*L)
E gene/SARS-CoV-2	426 bp	2.112	1.072	109.43	4.83 × 10^11^

**Table 2:** Repeatability of Ct value in each dilution concentration of synthesized RNA.

No.	Numbers (copy/*μ*L)	Mean (Ct value)	Cl 95% Ct value	Log copy Ct value 1	Log copy Ct value 2	Log copy Ct value 3	Average log copy	Average SD log copy	RSD % (log copy)
1	48,300	22.98	22.61-23.35	4.63	4.30	4.96	4.63	0.33	7.04
2	4,830	26.28	25.74-26.83	3.74	3.76	3.70	3.73	0.03	0.77
3	483	29.95	29.46-30.45	2.76	2.87	2.57	2.74	0.15	5.53
4	48.3	34.02	33.38-34.67	1.63	1.49	1.77	1.63	0.14	8.33

**Table 3:** Intermediate precision of Ct value per dilution concentration of synthesized RNA.

No.	Repeatability	Numbers (copy/*μ*L)	Mean (Ct value)	Log copy Ct value 1	Log copy Ct value 2	Log copy Ct value 3	Average log copy	Average SD log copy	RSD % (log copy)
1	Day 1	48,300	23.58	4.47	4.24	4.70	4.47	0.23	5.11
2	Day 2	48,300	22.50	4.76	4.63	4.89	4.76	0.13	2.80
3	Day 3	48,300	22.87	4.66	4.39	4.93	4.66	0.27	5.83
4	Day 1	4,830	27.15	3.36	3.31	3.82	3.50	0.28	8.11
5	Day 2	4,830	25.67	4.44	3.85	3.41	3.90	0.52	13.30
6	Day 3	4,830	26.03	3.53	3.80	4.07	3.80	0.27	7.15
7	Day 1	483	30.70	2.26	2.80	2.53	2.53	0.27	10.73
8	Day 2	483	29.28	3.19	2.92	2.65	2.92	0.27	9.31
9	Day 3	483	29.89	2.75	3.02	2.48	2.75	0.27	9.87
10	Day 1	48.3	34.79	1.42	1.56	1.29	1.42	0.14	9.56
11	Day 2	48.3	33.18	1.67	1.89	2.02	1.86	0.18	9.59
12	Day 3	48.3	34.10	1.52	1.61	1.69	1.61	0.08	5.24

**Table 4:** Accuracy test per each concentration for synthesized RNA.

Concentration levels	Ct values							Mean Ct value	Average log Ct value	Average SD log Ct value	RSD % Ct value
	1^st^ time	2^nd^ time	3^rd^ time	4^th^ time	5^th^ time	6^th^ time	7^th^ time				
80%	29.44	29.17	29.34	28.94	28.97	29.70	29.37	29.28	2.92	0.07	2.51
100%	29.00	29.42	29.23	29.16	29.36	29.71	29.79	29.38	2.89	0.08	2.70
120%	29.08	28.84	28.98	29.07	28.79	28.89	28.89	28.93	3.01	0.03	1.01

**Table 5:** The Ct values per each concentration for synthesized RNA.

No.	Numbers (copy/*μ*L)	Mean (Ct value)	Log copy Ct value 1	Log copy Ct value 2	Log copy Ct value 3	Average log copy	SD log copy	RSD % log copy
1	48,300	22.98	4.63	4.99	4.27	4.63	0.36	7.86
2	4,830	26.28	3.73	4.01	3.46	3.73	0.27	7.28
3	483	29.95	2.74	2.87	2.60	2.74	0.14	4.97
4	48.3	34.02	1.63	1.71	1.55	1.63	0.08	5.00

**Table 6:** Concentration of RNA extracted from the SARS-CoV-2 virus.

No.	Ct values			Log copy Day 1	Log copy Day 2	Log copy Day 3	Average log copy	SD log copy	RSD % log copy	Copy/reaction (5 *μ*L)
	Day 1	Day 2	Day 3							
1	19.94	20.27	20.10	5.46	5.37	5.41	5.41	0.04	0.83	259,226
2	20.26	20.03	20.08	5.37	5.43	5.42	5.41	0.03	0.61	255,577
3	19.69	20.19	20.55	5.52	5.39	5.29	5.40	0.12	2.17	258,182
4	23.80	23.53	24.06	4.41	4.48	4.34	4.41	0.07	1.63	25,852
5	23.61	23.58	23.78	4.46	4.47	4.41	4.45	0.03	0.66	28,003
6	23.59	23.60	23.80	4.46	4.46	4.41	4.44	0.03	0.72	27,895
7	26.78	26.97	27.19	3.60	3.55	3.49	3.54	0.06	1.57	3,514
8	26.96	27.09	27.07	3.55	3.51	3.52	3.53	0.02	0.54	3,369
9	27.13	26.91	27.29	3.50	3.56	3.46	3.51	0.05	1.48	3,238
10	27.87	28.65	28.24	3.30	3.09	3.20	3.20	0.11	3.32	1,607
11	28.26	28.39	28.55	3.20	3.16	3.12	3.16	0.04	1.25	1,441
12	27.89	28.99	28.45	3.30	3.00	3.14	3.15	0.15	4.75	1,455
13	29.17	30.65	27.46	2.95	2.55	3.41	2.97	0.43	14.61	1,276
14	29.17	30.13	29.26	2.95	2.69	2.92	2.85	0.14	5.05	738
15	29.17	30.09	29.09	2.95	2.70	2.97	2.87	0.15	5.26	774
16	30.41	31.77	31.55	2.61	2.24	2.30	2.38	0.20	8.32	261
17	30.21	32.30	31.43	2.67	2.10	2.33	2.37	0.29	12.06	268
18	30.27	31.49	30.96	2.65	2.32	2.46	2.48	0.17	6.71	315
19	34.23	33.16	34.09	1.57	1.86	1.61	1.68	0.16	9.39	50
20	34.51	33.66	34.04	1.50	1.73	1.63	1.62	0.12	7.16	42
21	33.81	33.70	34.56	1.69	1.72	1.48	1.63	0.13	7.80	44
22	36.42	35.78	36.23	0.98	1.15	1.03	1.05	0.09	8.48	11
23	37.03	37.19	37.34	0.81	0.77	0.73	0.77	0.04	5.47	6
24	38.03	38.19	38.34	0.54	0.50	0.46	0.50	0.04	8.46	3

**Table 7:** Summary of the criteria for synthesized RNA.

Criteria	Result	Required
LOD	~3 copies/reaction	
Linearity	0.98	0.98
Accuracy	1.01-2.51%	<25%
Precision		
Repeatability (intraprecision)	0.77-8.33%	
Interprecision	2.80-13.30%	

## Data Availability

The data used to support the findings of this study are available from the corresponding author upon request.

## Abbreviations

COVID-19: Coronavirus disease 19
LOD: Limit of detection
RT-qPCR: Quantitative reverse transcription PCR
SARS-CoV-2: Severe acute respiratory syndrome coronavirus 2
WHO: World Health Organization.
